# Calendar

**DOI:** 10.1155/S146392468300053X

**Published:** 1983


					Journal of Automatic Chemistry, Volume 5, Number 4 (October-December 1983), page 212
Calendar
March 1984
35th Pittsburgh Conference and
Exposition
To be held from 5 to 10 March 1984 in
Atlantic City, New Jersey, USA.
Further information from Mrs Linda
Brigos, Pittsburoh Conference, Depart-
ment J-212, 437 Donald Road, Pittsburoh,
Pennsylvania 15235, USA.
Editor's note
Organizers of conferences, seminars, etc.
should send details for inclusion in the
calendar as soon as the relevant infor-
mation is available and not later than three
months before the event.
November 1983
Second African, Mediterranean and Near
East Conference of Clinical Chemistry
To be held from 20 to 24 November in
Cairo.
Further information from the Congress
Secretariat, Second African, Medi-
terranean and Near East Congress of
Clinical Chemistry, 6 Porter Street, Baker
Street, London W1M 1H2.
December 1983
Quality Control of Wine
To be held on December at Harveys,
27 Pall Mall, London. Organized by the
Royal Society of Chemistry, Analytical
Division, Automatic Methods Group.
Further information from C. J. Jackson
Occupational Hygiene Laboratory,
403 Edgware Road, London NW2 6LN.
Analysis for Water
To be held on 6 December in London.
Further information from C. J. Jackson
(see above).
Robotics
To be held on 14 December in London.
Further information from C. J. Jackson
(see above).
January 1984
1984 Winter Conferences on Plasma
Spectroscopy
To be held from 2 to 6 January 1984 in
San Diego, USA.
Further informationfrom Dr R. M. Barnes,
Department Of Chemistry, GRC Towers,
University of Massachusetts, Amherst,
Massachusetts 01003, USA.
February 1984
Automation in the Cosmetics Industry
To be held on 15 February 1984 in
Northampton, UK.
Further information from C. .Jackson
(see aboe).
Automated Clinical Analysis
To be held on 7 March 1984 at Thames
Polytechnic in London.
Further information from C. J. Jackson
(see above).
April 1984
New Concepts in Quality Control
To be held on 2 and 5 April 1984 in
Cambridge, UK.
Further information from C. J. Jackson
(see above).
Analytica 84
To be held from 10 to 13 April 1984 in
Miinchen, FR Germany.
Further information from Dr Rosmarie
Vogel, Abteilungfir Klinische Chemie und
Klinische Biochemie in der Chirurgischen
Klinik Innenstadt der Universittt
Mfinchen, Nussbaumstrasse 20, D-8000
Miinchen 2, FR Germany.
16th Annual Symposium on Advanced
Analytical Concepts for the Clinical
Laboratory
To be held on 12 and 13 April 1984 at the
Hilton Hotel in Knoxville, Tennessee,
USA.
Further information from Debbie
Woodcock, Meetings Co-ordinator,
AACC, 1725 K. Street, Washington,
D.C. 20006, USA.
Royal Society of Chemistry: Annual
Chemical Congress
To be held from 16 to 19 April 1984 at the
University of Exeter, UK.
Further information from Dr John F.
Gibson, The Royal Society of Chemistry,
Burlington House, London WI VOBN.
Xllth International Congress of Clinical
Chemistry
To be held from 29 April to 4 May 1984 in
Rio de Janeiro, Brazil.
Further information from the Executive
Secretary, XIIth International Congress,
Rua Vicente Licinio 95, Cep 20270, Rio de
Janeiro, Brazil.
May 1984
Principles ofAutomation and Applications
of Robotics
To be held on 9 and 10 May 1984 at the
Scientific Societies Lecture Theatre,
23 Savile Row, London.
Further information from C. J. Jackson
(see above).
212
June 1984
2nd International Conference on
Chromatography and Mass Spectrometry
in Biomedical Sciences
To be held from 18-20 June 1984 in
Milan, Italy.
Further informationfromAlberto Frigerio,
Italian Group for Mass Spectrometry in
Biochemistry and Medicine, Via Eustachia
36, 20129 Milan, Italy.
Quality Control of Packaging: Food,
Beverages, Pharmaceuticals and Cos-
metics
To be held from 20 to 22 June 1984 in St.
Andrews, UK.
Further information from C. J. Jackson
(see above).
September 1984
Third European Symposium on Thermal
Analysis and Calorimetry
To be held from 9 to 15 September 1984 at
the Congress-Center-Casino Interlaken
in Interlaken, Switzerland.
Further informationfrom ESTAC 3 84, Dr
E. Marti, Im Langen Loh 181, CH 4054
Basel, Switzerland,
October 1984
2nd International Congress on
Automation and New Technology in the
Clinical Laboratory
To be held from 15 to 18 October 1984 in
Barcelona, Spain.
For further information contact 2nd
International Congress on Automation
and New Technology in the Clinical
Laboratory, IV Congreso National de la
Sociedad Espahola de Quimica Clinica,
Apartado de Correos 543, Barcelona,
Spain.
November 1984
14th Annual Symposium on the Analytical
Chemistry of Pollutants and, the 3rd
International Congress on Analytical
Techniques in Environmental Chemistry
To be held from 22-24 November 1984 at
the Palacio de Congresos in Barcelona,
Spain.
For further information contact 14th
Annual Symposium3rd International
CongressExpoquimia, Avda. Reina M".
Christina, Barcelona 4, Spain.
1985 meetings
IUPAC 1985
To be held from 9 to 13 September 1985 in
Manchester, UK.
Further information from The Royal
Society of Chemistry, Burlington House,
London W1 V OBN.

				

